# On the Treatment of Erysipelas by Silicate of Soda

**Published:** 1878-05

**Authors:** 


On the Treatment of Erysipelas by Silicate of Soda. This method has been employed specially by Dr. Alvarenga, of Lisbon, who credits it with grêat efficacy. His paper (an extract of which is gives in the *Journal Med. Chirurg. de Pesth*) is based on forty-eight cases of erysipelas of the scalp, face, and limbs, both fixed and erratic. He asserts that, with the help of this remedy, the disease does not last more than four or five days. The solution of silicate of soda used is the same which is employed in the manufacture of immovable apparatus. It is diluted with seven or eight times its weight of distilled water. It is very important to make a preliminary essay of this preparation with litmus paper; so long as it is acid, soda should be added to neutralize it. The solution must be spread over the affected parts, morning and night, with a pencil, and the surfaces must be allowed to dry in the air. 'At the end of four or five days, when the fever, œdema, and redness, have subsided, the use of the silicate of soda is suspended, and the parts affected are covered up with cotton-wool steeped in oil of sweet almonds.—*London Medical Record, Jan.* 15, 1878.

